# Surface plasmon-assisted microscope

**DOI:** 10.1117/1.JBO.23.6.060502

**Published:** 2018-06-22

**Authors:** Julian Borejdo, Zygmunt Gryczynski, Rafal Fudala, Chaitanya R. Joshi, Kathleen Borgmann, Anuja Ghorpade, Ignacy Gryczynski

**Affiliations:** a University of North Texas, Health Science Center, Department of Microbiology, Immunology and Genetics, Fort Worth, Texas, United States; b University of North Texas, Center for Commercialization of Fluorescence Technologies, Health Science Center, Fort Worth, Texas, United States; c Texas Christian University, Department of Physics and Astronomy, Fort Worth, Texas, United States

**Keywords:** surface plasmon coupled emission, microscopy, fluorescence

## Abstract

Total internal reflection microscopy (TIRF) has been a powerful tool in biological research. The most valuable feature of the method has been the ability to image 100- to 200-nm-thick layer of cell features adjacent to a coverslip, such as membrane lipids, membrane receptors, and structures proximal-to-basal membranes. Here, we demonstrate an alternative method of imaging thin-layer proximal-to-basal membranes by placing a sample on a high refractive index coverslip covered by a thin layer of gold. The sample is illuminated using the Kretschmann method (i.e., from the top to an aqueous medium). Fluorophores that are close to the metal surface induce surface plasmons in the metal film. Fluorescence from fluorophores near the metal surface couple with surface plasmons allowing them to penetrate the metal surface and emerge at a surface plasmon coupled emission angle. The thickness of the detection layer is further reduced in comparison with TIRF by metal quenching of fluorophores at a close proximity (below 10 nm) to a surface. Fluorescence is collected by a high NA objective and imaged by EMCCD or converted to a signal by avalanche photodiode fed by a single-mode optical fiber inserted in the conjugate image plane of the objective. The system avoids complications of through-the-objective TIRF associated with shared excitation and emission light path, has thin collection thickness, produces excellent background rejection, and is an effective method to study molecular motion.

## 1 Introduction

Total internal reflection fluorescence (TIRF) produces an evanescent wave, which decays exponentially with the distance from the coverslip. The most common application of TIRF in biological systems is through-the-objective geometry. In this geometry, the exciting light is passed through the periphery of a high NA objective to strike the glass at total internal reflection angle and induce an evanescent wave at the surface. Typically, the evanescent wave excites fluorophores within 100 nm from the glass. However, there are multiple undesirable events in the objective such as scattering by multiple lenses, reflections and refraction at surfaces, and autofluorescence of the lens material.^1^ The excitation and fluorescent light must travel twice through the objective. Another limitation of through-the-objective TIRF is that the excitation and emission light share the same path and that their separation is not perfect. Together, undesirable signals degrade the through-the-objective TIRF and produce abnormalities in the expected exponential decay.^2^^,^^3^ These problems have been addressed by through-the-prism and light guide geometries that minimize the interference of undesirable light and separate the excitation light from emission light path.^4^

A different solution to the through-the-objective TIRF is proposed here: it is realized by placing a sample on a thin metal film and illuminating it from the top with a laser beam through an aqueous medium. The excited fluorophores close to the surface couple (via the near-field interactions) to surface plasmons in the metal. Surface plasmons decouple on the opposite side of the metal film as a near-field radiation and emit in a directional manner. Fluorescence is collected with a high numerical aperture objective. A confocal aperture inserted in the objective conjugate image plane reduces lateral dimensions of the detection volume to a diffraction limit. The thickness of the detection volume is equal to the distance-dependent fluorescence coupling with surface plasmons.^5^ The thickness is further reduced by metal quenching of excited fluorophores at a close proximity (below 10 nm)^6^^,^^7^ to the surface. Here, we demonstrate that the reverse Kretschmann (RK) configuration has a number of demonstrable advantages over TIRF including the thinness of the observational volume, reduction of the background fluorescence, the ability to see only the fluorophores in a shallow volume residing 10 nm away from the surface, avoiding the subjective task of setting of the angle of incidence of the exciting beam [only the fluorescence that emerges at the surface plasmon coupled emission (SPCE) angle is collected by the objective], and the separation of the excitation and emission light paths.

We have shown previously that the application of the RK geometry is advantageous in fluorescence correlation microscopy,^8^^,^^9^ where a high rate of photon detection per molecule, low background, and large fluctuations of fluorescence associated with translational motion can be achieved. In this paper, we report improvements in the previous instrument, which involve inclusion of the beam expander to make the illumination more uniform, possibility of rotating the expander in Y−Z and X−Z directions to allow optimal illumination of a cell, the ability to regulate the intensity of the incident light, and inclusion of the adapter to adjust the distance between a sample and the illuminator. These advances combine to improve the quality of images of biological samples.

## 2 Materials and Methods

### 2.1 Chemicals and Solutions

Fluorescein-labeled fluorospheres (cat F8823, 2-μm diameter and cat F8803, 0.1-μm diameter) were from Thermo Fisher (Waltham, Massachusetts). They were supplied at $3.6\times 10^{13}/\mathrm{mL}$ and used at 20× dilution. All the other solvents were from Sigma (St. Louis, Missouri).

### 2.2 Preparation of Coverslips

A 50-nm-thick layer of gold was deposited on the high refractive index (sapphire) coverglasses from Olympus by EMF Corp. (Ithaca, New York). About 5-nm layer of silica was deposited on top of gold to protect it from oxidation. About 2-nm chromium undercoat was used as an adhesive background.

### 2.3 Sample Preparation

About 40-μL suspension of fluorospheres were placed on metal side of gold coverslips and covered (to reduce evaporation) with #1 22-mm square Corning (Corning, New York) coverslips. For control experiments, fluorospheres were placed on a glass rectangular coverslip (Corning #1 24×60  mm).

### 2.4 Microscopic Measurements

**Fig. 1:**
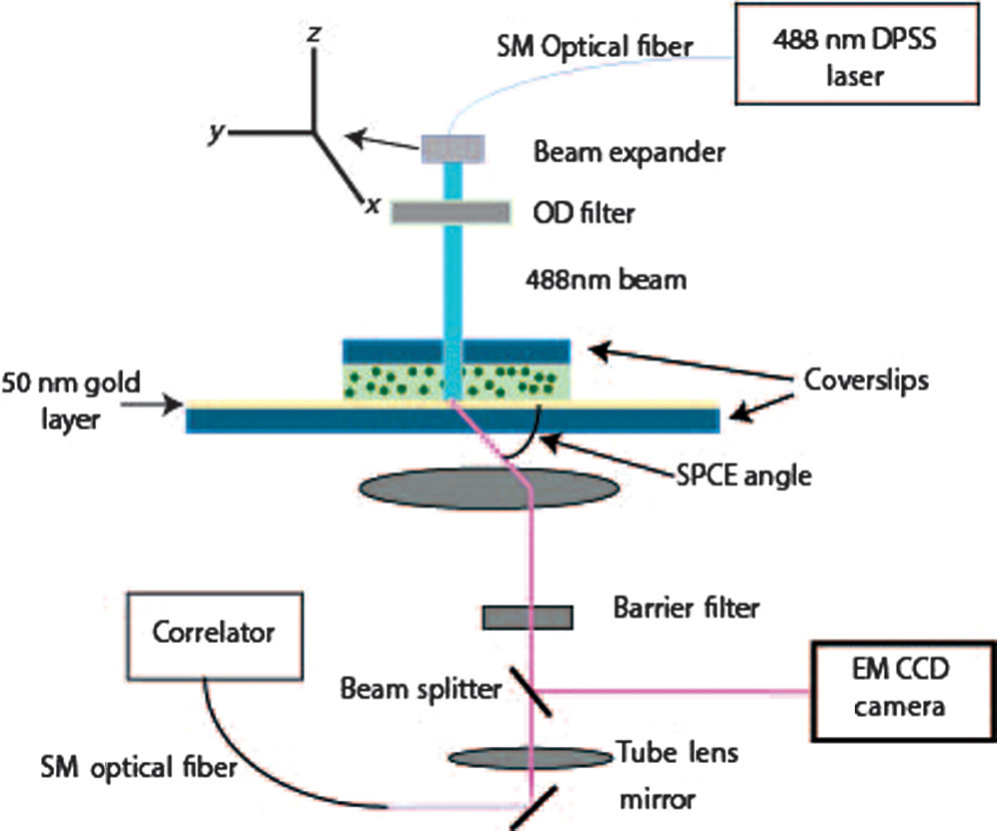
RK microscope and the principle of reverse Kretschmann measurements. Fluorescence from fluorophores near the gold surface produces surface plasmons in the metal. Fluorescent light couples with the plasmons to emerge at the bottom of the coverslip at a (SPCE angle; which is smaller than SPR angle). Only the fluorescence from molecules excited within ∼10 to ∼100  nm of the metal layer can penetrate the layer via plasmon resonance. Excitation light also gives rise to fluorescence from molecules outside this layer, but the emission is unable to penetrate the metal layer and enter the objective. This provides excellent background rejection. The fiber diameter was 3.5  μm, laser intensity was 50  μW, the intensity at the exit from single-mode fiber was 24  μW, the intensity entering objective was 3  μW, and the intensity at the exit from 60× objective was 0.9  μW.

The schematic diagram of the RK microscope is shown in Fig. 1. The beam of 488-nm light from a Melles Griot (Model 85-BCF-020-112) SPSS laser is carried by a single-mode optical fiber to a beam expander. The expanded beam (1 cm in diameter) is incident perpendicularly from above on a sample that is placed a gold coverslip that is resting on the moveable stage of an inverted Olympus IX71 microscope. The fluorescent light is emitted at SPCE angle and is collected by the objective (Olympus Apo 100×, 1.65 NA). The beam splitter inside the microscope directs the beam either to an electron multiplier CCD camera operating at −65°C (EMImage, Hamamatsu, Japan, pixel size 16×16  μm) attached to the side port of the microscope, or is projected onto a tube lens, which focuses it at the conjugate image plane of the microscope. The tip of an optical fiber (whose core acts as a confocal aperture) is inserted at this plane. An Avalanche photodiode (Perkin-Elmer SPCM-AQR-15-FC) collects light emerging from the aperture. The TTL signal from the diodes is fed to a correlator (Flex02-08D, Correlator Inc., Bridgewater, New Jersey), which acts as a data logger. The data are transferred to a PC that computes the time course of fluorescence, autocorrelation function (ACF), and histograms.

## 3 Results

### 3.1 Reverse Kretschmann Signal

**Fig. 2:**
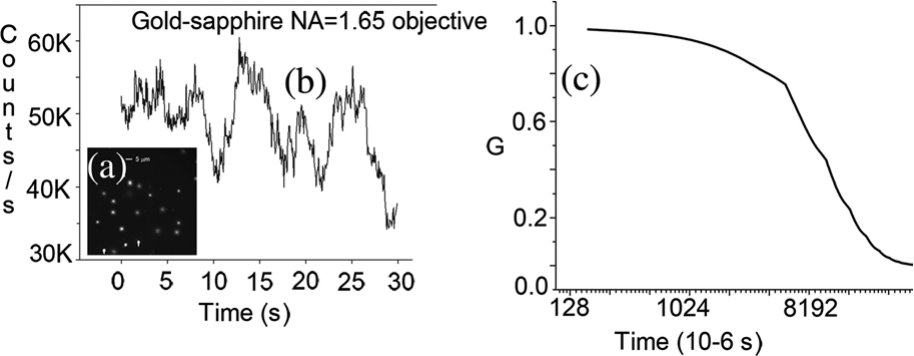
(a) 2-μm microspheres in RK. 1.65 NA ×100 Olympus objective, sapphire substrate, refractive index of immersion oil=1.78. (b) The intensity fluctuations caused by diffusion of 2-μm spheres through detection volume in RK experiment on gold substrate. (c) A typical ACF for 2-μm nanospheres. Fluorospheres were diluted 100 times to $3.6\times 10^{11}\text{ spheres}/\mathrm{mL}$.

Suspension of 2-μm diameter microspheres was diluted 10 times to $3.6\times 10^{12}\text{ spheres}/\mathrm{mL}$. The spheres were placed on a coverslip coated with gold. Figure 2(a) shows an image of the spheres on the gold-coated slide. Figure 2(b) shows a typical trace of intensity fluctuations. The intensities were measured in 160-μs intervals for 30 s. The fluctuations are caused by spheres entering and leaving the detection volume. As the thickness of the detection layer is 35 nm (see below), much smaller than the lateral dimension of diffraction limited laser beam (∼0.5  μm), the fluctuations in the intensity are contributed mostly by spheres translating in the Z-dimension. Figure 1(c) is the ACF of fluctuations.

It should be noted that TIRF correlation function has a pronounced “tail,”^8^ which is most likely caused by particles sticking to the glass. In contrast, the RK ACF has no “tail.” The particles also probably stick, but they are invisible because of metal quenching.

### 3.2 Background Rejection

**Fig. 3:**
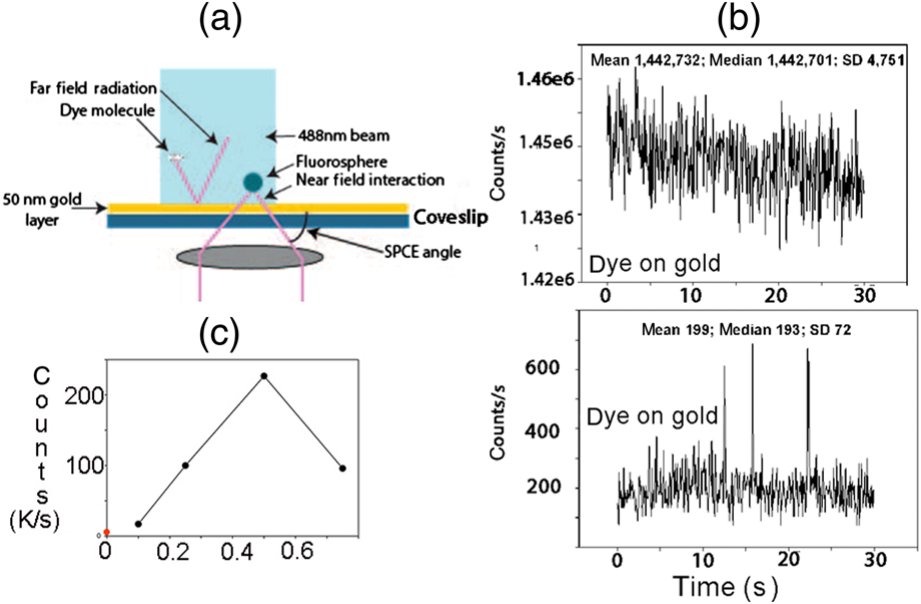
(a) The illustration shows how the contribution of the background is reduced. (b) Fluorescent signal from 0.26-mM Rhodamine 6G (in the absence of fluorospheres) on glass coverslip (top panel) is compared with fluorescence of the same concentration of dye on a gold-coated coverslip (bottom panel). Dye was illuminated by 543-nm laser and fluorescence was viewed through Olympus TRITC Filter Cube Set (#67-007). The detector settings were identical in (a) and (b). (c) Concentration dependence of rhodamine 110. The decrease in fluorescence at the highest concentration is due to inner filter and concentration quenching. The red dot indicates fluorescence of the coverslip.

Figure 3(a) shows that only fluorescence signal that enters metal layer through near-field interactions is able to penetrate the metal film. All near-field radiation enters the gold by coupling with surface plasmons and penetrates the metal surface to emerge at an SPCE angle. All far-field radiation is reflected by the metal film. Figure 3(b) compares the signal from 0.26-mM Rhodamine 6G on glass coverslip with the same dye on gold-coated coverslip. The background fluorescence is reduced by a factor of >7000. Figure 3(c) shows the dependence of signal on the concentration of the dye on the gold surface. The large background rejection applies only to the case when fluorescence originates 35 nm above the coverslip surface.

It should be noted that the method could also be applied in bulk (i.e., not in a microscope), where the suppression of background noise can be enhanced by taking advantage of directional and highly polarized character of SPCE signal.

### 3.3 Biological Samples

**Fig. 4:**
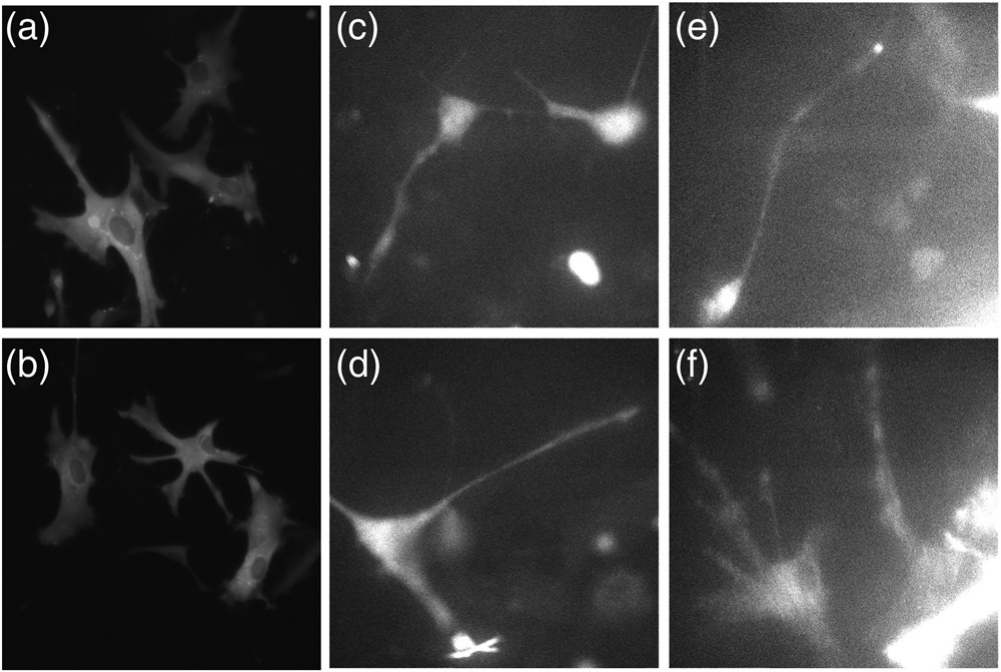
Images of human fetal astrocytes transfected with GFP. (a), (b) Live astrocytes on glass, viewed through ×20 objective. Micrographs were taken of each treatment at 400× original magnification using an Eclipse Ti-300 (Nikon) equipped with a black and white Luca R (Andor). Images were contrast enhanced; (c), (d) RK image of astrocytes on sapphire-gold coverslips viewed through Olympus FITC Filter Cube set (#67-004) contrast NOT enhanced. Average intensity of the background at the upper left corner: 35. (e), (f) TIRF images of astrocytes on glass coverslip, the same objective, and the same settings of the video camera as in (c), (d). Viewed through Olympus FITC Filter Cube set (#67-004). Average intensity of the background at upper left corner: 45 contrast NOT enhanced.

A typical image of cell monolayer of astrocytes is shown in Fig. 4. Astrocytes provide structural and metabolic support for brain neurons. Astrocytes transiently expressing GFP were cultured for 24 to 48 h before imaging. GFP binds nonspecifically to the body of a cell and cell membrane and to astrocyte processes.

The background rejection applies also to multilayers of cells. However, in the case when a sample consists of monolayers of cells, rejection is smaller because the background fluorescence is mostly contributed by the autofluorescence of glass and fluorescent glow of neighboring cells.

### 3.4 Reduced Thickness of Detection Layer

**Fig. 5:**
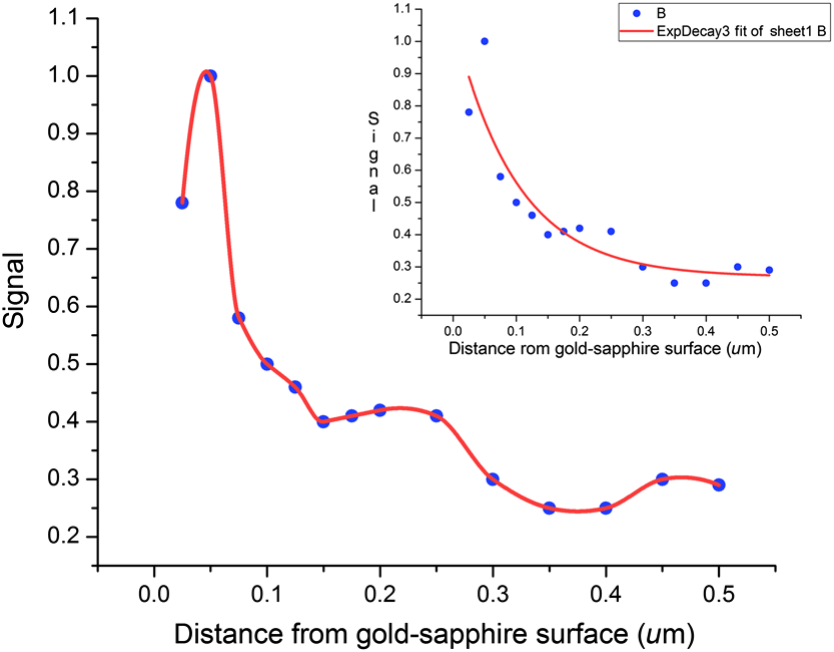
Thickness of detection layer in RK. The model of Ref. 8 was used to estimate the thickness. The theoretical approach to fitting the curves is the same as used in Ref. 9. It is an extension of Hassler et al theory.^11^ It uses linear combinations of exponentials axially and a Gaussian laterally. The maximum intensity at emission into the sapphire prism is at ∼35  nm. It is assumed that the exciting field is continuous in time and weak enough such that the time between excitations is much longer than the time between excitation and emission of the fluorophore. This means that the average number of photons emitted per unit time and therefore the average total emitted power does not depend on the lifetime; they only depends on the average time between excitations. The refractive indices of the metals used in the calculations are interpolated from Ref. 12. Inset: the same curve fitted with a single exponential. 1/e is 200 nm. Normal incidence. Metal is 50-nm layer of gold.

The emission into the objective in RK originates from a volume that is thinner than in TIRF. Figure 5 (based on calculations in Ref. 9) shows that emission into the objective peaks around 35 nm. Also, in RK the fluorescence is quenched near the gold surface. The detected emission comes from a thin detection volume slightly above the metal surface. The rejection of the emission of fluorophores near the metal surface should be better for small fluorophores on the proximal surface of a cell than for spherical fluorescent beads employed here. In TIRF, there is no such rejection of emission near the surface.

The thickness could not be determined experimentally by varying coating thickness and observing molecules immobilized or evaporated on this coating. Application of thin coating on top of gold results in an emission at several angles in the glass substrate, with either s or p polarization. This complex pattern of directional and polarized emission appears to be due to optical waveguide effects occurring when the sample thickness becomes comparable with the emission wavelength.^13^^,^^14^ For example when layer is 290-nm thick, SPCE is observed at two angles, with different s or p polarization for each angle. Even more complex pattern is observed thicker films. The multiple rings of SPCE and the unusual s-polarized emission are consistent with the expected waveguide modes in the silver–PVA composite film.

## 4 Discussion

The X−Y dimension in RK is no better than in TIRF. However, the axial dimension in RK is better than in TIRF. The thin detection volume results from a combination of two processes. First, SPCE coupling decays exponentially from the surface. Second, fluorophore is quenched in close proximity to the metallic surface.^6^^,^^7^ This metal quenching effect displaces observable fluorescence 5 to 10 nm from the surface. Simulations shown in Figure 5 indicate that the active range for the fluorescence signal can be as small as 35 nm.

The background rejection of RK is superior to TIRF because only the SPCE emission couples through the metal film and is collected by the objective. Free space emission fluorescence does not couple and is rejected by the metallic surface. The background fluorescence in Fig. 3(b) is reduced by a factor of >7,000.

Because the axial dimension of RK is <200  nm is at least two times smaller than the lateral dimension (∼0.5  μm), the major fluctuations arise from molecules moving axially. This is important in the study of kinetics of ligand–receptor interactions in cell membranes and of adsorption of small molecules at solid/liquid interfaces to understand the mechanisms of regulation of the initiation of cell signaling.^15^ Because of the thinness of detection volume, the fluctuations contributing to ACF of Fig. 3(b) originate from a single fluorosphere [the number of contributing molecules $N=\underset{\tau \to 0}{\frac{1}{2}\;\lim}\frac{1}{G(\tau )-1}=1/G(0)$].

In the case of biological samples lying flat on the surface, quenching will be more efficient than for spheres that may not be able to penetrate well into the quenched region near the metal surface.

It should be emphasized that added advantage of RK over TIRF is that, because of quenching, the fluorescence signal is contributed by fluorophores at least 10 nm away from the surface.^9^ As 10 nm is a typical thickness of cell membrane, the RK avoids monitoring events occurring at a cell membrane and looks only at the fluorophores inside a cell. In cells like astrocytes, kidney cells, rat neurons, or fibroblasts,^16^ the distance of the major part of the membrane is separated by >50  nm from the surface and the quenching offers no benefit. However, in cells in which the distance between a cell and a substrate is small (∼10  nm), such as erythrocyte ghosts on polylysine^17^ quenching offers substantial benefit because it allows to probe deep inside a cell.

In summary, advantages of RK over TIRF include:

a. The observational volume is shallower than TIRF because of the SPCE effect and quenching of fluorescence by the metal. The height of the detection volume is ∼35  nm. This makes observational volume small enough to image a single molecule.
b. The excitation and emission paths are different so image quality is not degraded by the imperfections in a dichroic mirror.
c. The quality of image is less degraded by imperfections in an objective because light passes through it only once.
d. The background contributed by fluorescence emitted into free space is reduced by at least two orders of magnitude. Only fluorescence that enters metal layer at SPCE angle is able to penetrate the metal film. All other emission is reflected by the metal film.

Disadvantages of RK over TIRF include:

a. Because SPAM angle is larger than TIRF angle, a high (NA=1.65) objective, sapphire coverslips, and high refractive index (toxic) immersion oil are needed for RK, but only relatively high (NA=1.45) objective, glass coverslips, and nontoxic immersion oil is sufficient for TIRF.
b. KR offers no significant advantages over TIRF as far as fluorescence collection efficiency and brightness are concerned.^18^ (i.e., it is less sensitive than TIRF)
